# Lesion volume and location can be estimated by analysis of the new NIH Stroke Scale picture description

**DOI:** 10.1371/journal.pone.0337429

**Published:** 2025-12-03

**Authors:** Melissa D. Stockbridge, Voss Neal, Shun Liu, Lindsey Kelly, Isidora Diaz-Carr, Joseph S. Kang, Andreia V. Faria, Argye E. Hillis

**Affiliations:** 1 Department of Neurology, Johns Hopkins University School of Medicine, Baltimore, Maryland; 2 Department of Radiology, Johns Hopkins University School of Medicine, Baltimore, Maryland; 3 Department of Physical Medicine and Rehabilitation, Johns Hopkins University School of Medicine, Baltimore, Maryland; 4 Department of Cognitive Science, Krieger School of Arts and Sciences, Johns Hopkins University, Baltimore, Maryland; Museo Storico della Fisica e Centro Studi e Ricerche Enrico Fermi, ITALY

## Abstract

In January 2024, the picture stimuli used to elicit language on the NIH Stroke Scale were updated. Descriptions elicited using the now-retired *Cookie Theft* picture are informative regarding the size and location of stroke. An important dimension of validation for the new stimuli is to demonstrate that the language patients produce when describing the new *Precarious Painter* picture that is now part of the NIH Stroke Scale is similarly informative. Participants included 62 patients with acute ischemic stroke (24 left and 38 right) compared with 10 control participants hospitalized following transient ischemic attack. Descriptions of the *Precarious Painter* image were analyzed for content and structure. Bilateral regions of interest were selected based on their role in language processing, and lesion volume and location were measured on diffusion-weighted imaging. Analyses of variance were used to contrast discourse performance across groups, and correlations between discourse characteristics and proportion of region lesioned were calculated. Patients with left hemisphere strokes included less content from the right side of the picture, F(2) = 7.4, p = 0.001, η^2^_P_ = 0.19, than those in the other groups. Informational efficiency had a strong inverse relationship with extent of lesion to the left, but not right, IFG. Correlations between discourse variables and lesion locations were grossly consistent with those previously reported and evidenced relationships between discourse variables and the bilateral extended language network. Features of picture description that may be appraised without specialized training in communication, such as how many things a patient includes, their location, and the inclusion of inferences or summarizing statements in the description, provide insight into stroke location and severity. The samples elicited using the new *Precarious Painter* stimulus perform similarly to those elicited using the retired *Cookie Theft* stimulus.

## Introduction

The NIH Stroke Scale is a valuable tool for evaluating acute stroke [1–3] and has been associated with infarct characteristics [4] and long-term functional outcomes. [5–7] Patients receiving the NIH Stroke Scale are asked to describe a pictured scene in order for the administrator to identify the presence and severity of aphasia, or language disruption. Patients score a “0” for subjectively “normal” language and “3” if they are mute. The scores in between parse patient communication as a function of the administrator’s level of burden in the interaction. If the patient is producing any oral communication, but the burden of interpretation falls predominantly or exclusively on the administrator, then the patient receives a “2,” and any present lesser disruption is rated a “1.” From the perspective of clinical specialists in language, the NIH Stroke Scale rating of aphasia is coarse. The picture description task also may inform the identification of neglect or inattention, which is a relatively common consequence of stroke that occurs in nearly two in five patients after right hemisphere stroke and one in five after left hemisphere stroke [8]. While hyperacute neuroimaging is increasingly the norm for stroke care around the world, information gleaned from the NIH Stroke Scale may have particular clinical utility in care contexts when this imaging is not available.

In January of 2024, the picture stimulus used to elicit language on the NIH Stroke Scale, formerly the *Cookie Theft*, was updated to a more modern image, the *Precarious Painter,* after nearly 30 years of near universal, international use [9]. A decade ago, authors Agis *et al*. demonstrated that quantitative analysis of the *Cookie Theft* picture 1) was feasible and reliable (92.5% point-to-point percent agreement) among physicians treating stroke without a specialization in communication or language disorders following a < 10 minute training in the procedure, 2) added <5 minutes to the administration of the NIH Stroke Scale, and 3) provided valuable information about lesion location and size beyond what is captured on the remainder of the Scale [10]. An important dimension of this added information was that it captured a wider array of capacities specifically considered to be associated with the right hemisphere. Previous studies have demonstrated that the standard administration of the NIH Stroke Scale is biased against detecting right hemisphere strokes; the volume of lesion to the right hemisphere is underestimated relative to the volume of lesion in the left hemisphere [4,11]. NIH Stroke Scale scores predict the location of damage within the right hemisphere more poorly than within the left hemisphere [12]. This may be due to fewer cognitive functions associated with the right hemisphere being included on the assessment [13]. Nonetheless, this bias is an important caveat to interpretation of the standard NIH Stroke Scale administration that adding quantitative analyses of picture description of the *Cookie Theft* appeared to mitigate.

The quantitative analysis used by Agis *et al*. relied upon *content units* (CUs), an inventory of previously-established objects, actions, and concepts within a given picture that are counted based on their location to the left or the right of the image [14]. *Cookie Theft* total CUs significantly correlate with lesion volume to the left and the right; however, examining the rate or efficiency of producing content (syllables/CU) resulted in stronger relationships [10]. CUs and syllables/CU also well associated with other behavioral assessments of language, valuable for predicting recovery from acute performance [15], and may even be more sensitive than the most common measures for detecting language difficulty [16].

A confident shift in clinical practice necessitates re-examining psychometric properties of the NIH Stroke Scale picture description in the context of the new *Precarious Painter* image. The first step already has been taken: establishing healthy normative data [9]. Clinical normative data collection is ongoing. The aim of this investigation was to revisit the basis of Agis *et al.* with the *Precarious Painter* to determine whether quantitative analysis of NIH Stroke Scale picture description contributes valuable information about the volume and location of the acute infarct.

## Materials and methods

Participants included 62 English-speaking adult patients within 7 days of ischemic stroke onset (24 left hemisphere and 38 right hemisphere) compared to 10 similarly aged control participants hospitalized following TIA. Patients were recruited through an ongoing longitudinal observational trial of post-stroke recovery from April 1, 2023 until the desired enrollment was reached July 30, 2024. The use of human subjects for the study was approved by the Johns Hopkins University School of Medicine Institutional Review Board-2 (NA_00042097) and all participants or their legally-authorized representatives provided written consent. The work is supported by NIH/NIDCD R01 DC05375. Demographic information was derived from the electronic medical record, where evidence of acute hemispheric ischemic stroke was confirmed (or ruled out, for TIA patients) with clinical-quality MRI. Individuals with a prior history of neurological disease affecting the brain other than stroke (e.g., dementia, brain tumor, multiple sclerosis), known hearing loss, or uncorrected visual loss were excluded. Those who were pregnant, endorsed severe claustrophobia, or had a pacemaker or ferromagnetic implant were excluded, as they were unable to receive MRI. Data are described in accordance with the Strengthening the Reporting of Observational Studies in Epidemiology (STROBE) initiative [17]. Self-described demographic information was collected, including age (in years), sex, race, ethnicity (Hispanic/Non-Hispanic), and educational attainment.

### Picture description procedures and data extraction

Picture descriptions were elicited bedside during acute hospitalization by presenting the *Precarious Painter* image on a standard 8.5” x 11” sheet of white paper and asking patients to “Please describe everything you see happening in this picture using complete sentences if you can” (see **Table 1** for examples). There were no time limits placed on responses, though most responses were completed in under 3 minutes as indicated by the speaker. Responses were analyzed for total CUs, ratio of left:right CUs, syllables/CU (a measure of informational efficiency), and percent interpretive CU. Where no content was described, syllables were divided by 0.1. Although Agis *et al.* demonstrated that modest additional training results in highly reliable simultaneous inventorying of CUs during *live* administration of the NIH Stroke Scale, picture descriptions were audio recorded for the present study to facilitate later syllable counts. A previously-established inventory of CUs commonly produced by normal adult participants without cognitive or communication diagnoses, [9] similar to the inventory available for the *Cookie Theft* [14,18], was used to identify CUs.

**Table 1:** Example picture descriptions by acute patients with left and right hemisphere stroke.

Left hemisphere stroke	“Hm a kid uh going up the ladder. A mouse running over the floor chasing uh the dog chasing a m- uh mess uh m- chaos.
Right hemisphere stroke	“The picture’s crooked on the the painting is crooked. The person is surprised or shocked and I’ll say it looks like they’re painting. Did you know I was an American Indian? My grandma was an American Indian in Cumberland, Maryland.”

### MRI processing and analysis

Clinical-quality MRIs (3 T) conducted during routine care were retrieved from each participant’s medical record. Lesions were identified in diffusion-weighted images using the deep learning-based detection and segmentation pipeline described in Liu et al., [19] in which lesions are segmented with 3D DAGMNet. After this process was completed, results were visually inspected for quality prior to analysis. The bilateral regions of interest were selected based on their role in language processing: inferior frontal gyrus (IFG; pars opercularis, pars orbitalis, pars triangularis), superior temporal gyrus (STG) and pole, middle temporal gyrus (MTG) and pole, inferior temporal gyrus (ITG) supramarginal gyrus (SMG), fusiform (Fu), and angular gyrus (AG).

### Statistical analysis

Statistical analyses were done in R 4.4.3 and SPSS. Power analyses were done with G*Power 3.1.9.7. Sample size was based on that of Agis *et al.*, who reported on 67 patients and found effect sizes ranging from 0.6–0.8. An *a priori* power analysis for correlation demonstrated that a sample size of 62 patients would allow for the detection of an effect size of 0.6 given an alpha error probability of 5%. Pearson correlations were calculated between quantitative discourse measures and ROIs separately for left and right hemisphere stroke.

We tested the hypothesis that picture description analysis provides useful information about lesion volume independently of age using multivariable regression analyses. Total infarct volume was the dependent variable, while age, total CU, syllables/CU, percent interpretive CU, and left:right CU were entered as independent predictors. The multiple regression analysis of the complete sample in Agis et al. found an effect size of f^2^ = 1.17 based on a 5-predictor multiple regression model. Given our multiple regression model had fewer predictors than were used in Agis et al., an *a priori* power analysis found that a noncentrality parameter of λ = 25.83 results in a critical F value of 2.96. The sample size needed for an actual power of 0.96 was 22 participants, well below the number available to complete the present analysis.

In order to examine whether *Precarious Painter* description contributed information about lesion site, linear regression analyses were run to determine which lesion sites were associated with each picture description variable when controlling for age. Dependent variables were total CU, syllables/CU, left:right CU, and percent interpretive CU. These analyses were run separately for left and right hemisphere stroke patients. Independent variables for each multivariable regression were total lesion volume, age, and damage to each ROI. The ROIs for each discourse variable were selected by determining those that significantly correlated with the variable tested. Agis et al. used a binary coding for ROI involvement (>5%) in regression models. However, in the current sample drawn from more recent patients, lesion volumes were smaller on average, and there was concern that this transformation would result in considerable loss of meaningful signal. Consequently, the decision was made to instead use % of ROI lesioned in the regressions.

Anonymized patient data will be available on the Vivli repository within one year of publication. Further descriptive data are available upon request to the authors, subject to review by the Johns Hopkins University School of Medicine Institutional Review Board resulting in a formal data sharing agreement.

## Results

**Fig 1:**
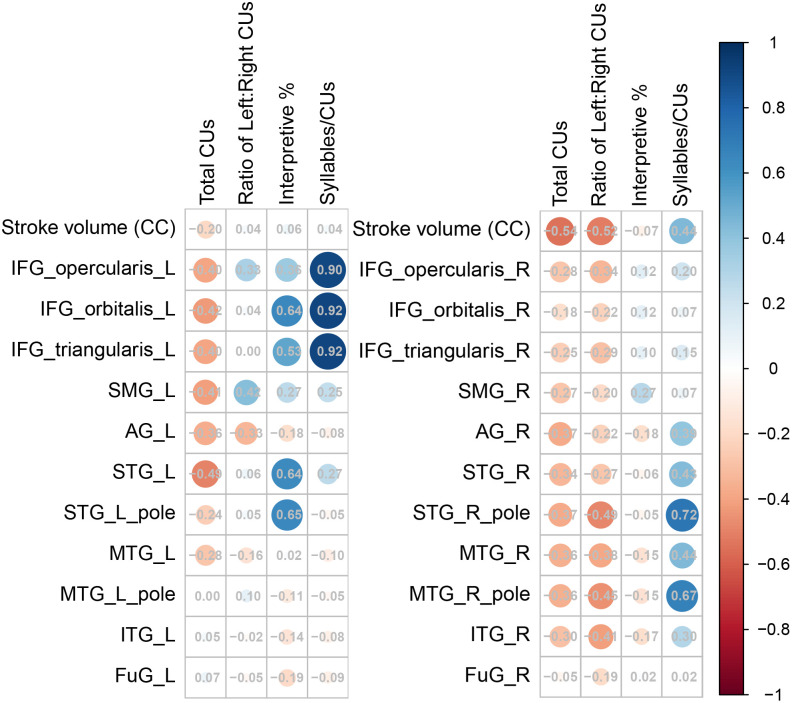
Lesion size and localization correlation with discourse measures. Correlations were visualized using a color and size matrix with hue representing negative (warm) and positive (cool) direction and both circle size and value related to strength of association. Left and right hemisphere stroke patients were considered independently. Those with left hemisphere strokes showed negative correlations between lesion extent within the distributed language network ROIs and total content and positive correlations between lesion to these ROIs and interpretive content. Strong positive associations were noted between informational efficiency and the inferior frontal gyrus (IFG); using more syllables to convey each item (less efficiency) was associated with greater extent of lesion. Those with right hemisphere strokes also demonstrated similar effects of damage to ROIs in the contralateral distributed language network, though efficiency was less associated with frontal damage and more associated with homologues to classical language areas in the STG and MTG.

Groups were similar in age, sex, education, and overall stroke volume (**Table 2**). Groups with stroke also were similar in the amount of content they produced (F(60) = 1.36, p = 0.25), percent interpretive content (F(56) = 0.59, p = 0.45), and the efficiency with which they produced content, measured in syllables/CU (F(60) = 1.3, p = 0.26; **Table 3**). However, groups differed in the ratio of left:right content, F(56) = 13.4, p < 0.001. Correlations between discourse measures and lesion volume at each location differed depending on the lateralization of the lesion (**Fig 1**).

**Table 2:** Sample characteristics.

	Left hemisphere	Right hemisphere	TIA
N	24	38	10
Age (years)	66 ± 14	67 ± 11	57 ± 14
Sex (F:M)	8:16	13:25	2:8
Race (Black:White:Other)	15:9:0	19:18:1	5:2:0
Education (years)	14 ± 3	15 ± 3	13 ± 2
Stroke volume (cc)	20 ± 29	28 ± 32	

**Table 3:** Discourse characteristics.

	*Precarious Painter*			*Cookie Theft* (from Agis *et al.*)		
	Left	Right	TIA	Left	Right	TIA
Content (CUs)	13.2 ± 7.4	15.8 ± 9.8	20.3 ± 5.7	7.3 ± 4.0	9.9 ± 5.3	16.0 ± 3.7
Left:Right*	1.8 ± 1.2	1.0 ± 0.5	1.1 ± 0.5	1.5 ± 1.1	0.8 ± 0.6	1.2 ± 0.4
Interpretive (%)	6.0 ± 6.6	4.8 ± 5.6	1.4 ± 1.1	40 ± 24	33 ± 22	39 ± 10
Syllables/CU	31 ± 111	10 ± 10	7 ± 3	12 ± 13	9 ± 7	6 ± 2

### Predicting total lesion volume from discourse

In a multivariate regression, total infarct volume was the dependent variable, while age, total CU, syllables/CU, and percent interpretive CU were entered as independent predictors. The model was significant, F(4, 53) = 2.6, p = 0.045, R^2^ = 0.165, with discourse measures alone predicting just over 16% of the variance in total lesion volume regardless of stroke lateralization. Coefficients are presented in **Table 4**.

**Table 4:** Predicting total lesion volume from discourse.

	B ± SE [95% CI]	β	Sig.
Constant	63.0 ± 25.9 [10.9, 115.0]		0.019
Total CU	−1.4 ± 0.5 [−2.4, −0.5]	−0.4	0.005
Interpretive CU %	−0.2 ± 0.7 [−1.6, 1.1]	−0.004	0.977
Syllables/CU	−0.02 ± 0.7 [−1.3, 1.3]	−0.044	0.736
Age	−0.02 ± 0.3 [−0.9, 0.4]	−0.095	0.457

### Using discourse measures to inform lesion location

Regression analyses were pursued separately for patients with left and right hemisphere stroke.

Among those with left hemisphere stroke, total content correlated significantly with the lesioned proportion of the IFG pars orbitalis (p = 0.039), SMG (p = 0.046), and STG p = (0.015). Ratio of left:right content correlated only with the SMG (0.050). Percent interpretive content and efficiency both were strongly correlated with frontal structures, as anticipated. Interpretive content correlated significantly with the IFG pars orbitalis (p = 0.001) and pars triangularis (p = 0.012), STG (p < 0.001), and specifically the STG pole (p < 0.001). Efficiency correlated significantly with only the IFG pars opercularis (p < 0.001), pars triangularis (p < 0.001), and pars orbitalis (p < 0.001).

For each discourse variable, age and total lesion volume was included in the linear regression with the identified ROIs. Results including standardized betas in order to facilitate comparisons across models are summarized in **Table 5**. Unstandardized betas are reported in Appendix A. Of the four models, only the model predicting informational efficiency (syllables/content unit) was significant, F(5, 18) = 30.1, p < 0.001, accounting for 89% of the variance. Percent damage to the IFG pars opercularis was a significant independent predictor, t = 2.3, p = 0.04.

**Table 5:** Summary of models predicting discourse variables from ROIs in left hemisphere stroke.

	Age	Vol	IFG Orb	IFG Tri	IFG Oper	SMG	STG	STG Pole	R^2^	F
Total CUs	−0.02	−0.1	−0.2			−0.2	−0.3		0.32	F(5, 18) =1.71, p = 0.18
Left:Right	−0.2	0.2				0.4			0.25	F(5, 18) = 1.95, p = 0.16
Interp.	−0.1	−0.3	−8.7	2.8			−0.2	7.3	0.5	F(5, 18) = 2.47, p = 0.07
Syll/CU	−0.1	−0.1	0.1	0.5	0.4*				0.89	F(5, 18) = 30.1, p < 0.001

*p < 0.05. Standardized betas are reported. CU: Content Unit. Interp.: Percent interpretive content. Syll/CU: Syllables/Content units. Vol: Total lesion volume. IFG Orb: IFG pars orbitalis. IFG Tri: IFG pars triangularis. IFG Oper: IFG pars opercularis. See Appendix A for unstandardized betas and confidence intervals.

Among those with right hemisphere stroke, total content correlated significantly with the lesioned proportion of the STG (p = 0.04), MTG (p = 0.04), and AG (p = 0.02). Ratio of left:right content correlated with the MTG (p = 0.04) and ITG (p = 0.01). Informational efficiency was correlated with STG (p = 0.01), STG pole (p < 0.001), MTG (p = 0.01), MTG pole (p < 0.001), and AG (p = 0.02). Proportion of interpretive content was not significantly associated with any ROI considered independently, and the planned model was not pursued.

Results of the linear regressions are included in **Table 6**. The model of total content units was unable to be fully expressed, as unstandardized coefficients were estimated at zero for all ROIs included in the model. Standardized coefficients are provided. The model predicting the ratio of left:right content among patients with right hemisphere stroke was significant, F(4, 31) = 3.41, p = 0.02, and explained 31% of the variance in the dependent variable. However, none of the ROIs were significant predictors. As was observed among those with left hemisphere stroke, the model predicting informational efficiency also was significant, F(7, 30) = 22.88, p < 0.001, and predicted 84% of the variance. Total lesion volume and lesioned proportion of the STG pole (t = 2.2, p = 0.04), MTG (t = 3.5, p = 0.001), and MTG pole (t = 4.5, p < 0.001) all were significant independent predictors.

**Table 6:** Summary of models predicting discourse variables from ROIs in right hemisphere stroke.

	Age	Vol	STG	STG Pole	MTG	MTG Pole	ITG	AG	R^2^	F
Total CUs	−0.2	−0.6	−0.01		0.1			−0	0.27	2.36 p = 0.06
Right:Left	−0.3	−0.4			0.1		−0.2		0.31	3.41 p = 0.02
Syll/CUs	0.1	0.4*	0.6	−1*	−1*	1.8*		0.1	0.84	22.88 p < 0.001

*p < 0.05. Standardized betas are reported. CU: Content Unit. Interp.: Percent interpretive content. Syll/CU: Syllables/Content units. Vol: Total lesion volume. IFG Orb: IFG pars orbitalis. IFG Tri: IFG pars triangularis. IFG Oper: IFG pars opercularis. See Appendix A for unstandardized betas and confidence intervals.

## Discussion

The description of the previous picture used in the NIH Stroke Scale was established as a valuable source of information regarding lesion extent and location not captured by a standard administration of the assessment. The aim of the present work was to bolster clinical confidence in the new *Precarious Painter* image included in the NIH Stroke Scale by showing that quantitative analysis of NIH Stroke Scale picture description contributes valuable information about the volume and location of the acute infarct. Features of picture description, such as the overall length and the quantity, quality, and location of content described, provided insight into stroke location and severity.

The samples elicited using the new *Precarious Painter* stimulus performed similarly to those elicited using the retired *Cookie Theft* stimulus in their relationship to lesion extent and location. As in the analysis of the *Cookie Theft,* lesion volume correlated negatively with total CU in right hemisphere stroke, though the relationship was weaker in left hemisphere stroke, likely because the most severe left hemisphere stroke patients were unable to provide informed consent for the study (and legally authorized representatives were often unavailable to do so), resulting in fewer participants with large left hemisphere strokes. As in the *Cookie Theft*, lesion volume negatively correlated with the ratio of left:right content only in right hemisphere stroke. Probing this further, we found that among these participants, this was driven by the relationship between the left:right ratio and lesion to the STG and MTG poles.

In contrast with the correlational results, discourse measures alone provided a poor prediction of overall lesion volume. However, examining the relationships between performance and lesion localization was more informative. Among those with left hemisphere stroke, the amount of content was associated with classical areas within the left hemisphere temporal language areas. Efficiency and interpretive content were associated with more frontal lesions. However, this pattern was quite different in those with right hemisphere stroke. Informational efficiency had a strong inverse relationship with extent of lesion only in the left, but not right, IFG. Lesions to the right hemisphere extended language network in particular were associated with informational efficiency. This likely reflects that the underlying cause of inefficiency is different between the two groups. Patients with left hemisphere stroke who are able to participate in a discourse task engage in strategies to circumvent word-finding difficulties, resulting in more explanation and filler words (e.g., “um,” “uh”). Both of these would result in higher numbers of syllables in patients whose IFG was lesioned. In contrast, those with right hemisphere strokes may only have demonstrated inefficiency in communicating the content of their message if their stroke was very large and disrupted their distributed bilateral language network.

Interpretive content (e.g., referring to certain events using causal terminology versus viewing different events in the picture independently of one another) is less studied relative to examining total content, efficiency, and left:right lateralization. One interpretation is that their inclusion may relate to network level disruption rather than focal disruption. This is an area where future work is indicated, as inferences are a crucial part of real-world discourse level language.

A key interest in this investigation was to examine whether enriched analyses of picture description would provide information about right hemisphere stroke location. Extent of lesion to the right STG pole, MTG, and MTG pole predicted 84% of the variance in informational efficiency among those with right hemisphere stroke. In contrast to the NIH Stroke Scale, which correlates predominantly with motor regions, the picture description provides complementary information about lesion location.

One limitation of this study and the study of the *Cookie Theft* image is that the samples were not obtained in the hyperacute stage of stroke. Patients were evaluated as soon as possible after admission for acute ischemic stroke, nearly always within 72 hours of onset. The descriptions were not from the initial NIH Stroke Scale (generally administered by the neurology resident on call without obtaining consent for research or recordings). Future studies should examine the hyperacute period specifically using the initial NIH Stroke Scale. Nevertheless, these findings complement the previous investigation and provide important validation that further supports the adoption of the modernized images.

## Supporting information

S1 File Appendix A. (DOCX)
