# Supplementary material for: Lesion volume and location can be estimated by analysis of the new NIH Stroke Scale picture description
Source: PLoS One. 2025 Dec 3;20(12):e0337429. doi: 10.1371/journal.pone.0337429 (PMC12674517; doi:10.1371/journal.pone.0337429)
Supplement: S1 File — (DOCX) [file pone.0337429.s001.docx]

Appendix A: Unstandardized beta values associated with models predicting discourse from ROI lesioned.

**Left hemisphere stroke**

|  | Constant | Age | Vol | IFG Orbitalis | IFG Triangularis | IFG Opercularis | SMG | STG | STG Pole |
| --- | --- | --- | --- | --- | --- | --- | --- | --- | --- |
| Total CUs | 15.9+7.4  [0.3, 31.6]* | -0.01+0.1  [-0.2, 0.2] | -0.03+0.08  [-0.2, 0.1] | -0.005+0.007  [-0.02, 0.01] |  |  | -.004+0.004  [-0.01, 0.004] | -0.005+0.006  [-0.02, 0.006] |  |
| Left:Right CUs | 2.5+1.1  [0.1, 4.9] | -0.02+0.02  [-0.05, 0.02] | 0.01+0.01  [-0.01, 0.04] |  |  |  | 0.001+0.001  [0.0001, 0.002] |  |  |
| Interpretive CU % | 9.3+6.4  [-4.4, 23.0] | -0.1+0.1  [-0.3, 0.2] | -0.1+0.1  [-0.2, 0.1] | -0.5+0.7  [-2.0, 1.0] | 0.1+0.2  [-0.2, 0.4] |  |  | -0.005+0.5  [-0.1, 0.1] | 0.1+0.1  [-0.1, 0.3] |
| Syll/CU | 28.3+44.0  [-64.2, 120.9] | -0.4+0.7  [-1.8, 1.0] | -0.3+0.4  [-1.2, 0.7] | 0.06+0.2  [-0.3, 0.4] | 0.1+0.1  [-0.1, 0.4] | 0.05+0.02  [0.004, 0.1]* |  |  |  |

**Right hemisphere stroke**

|  | Constant | Age | Vol | STG | STG Pole | MTG | MTG Pole | ITG | AG |
| --- | --- | --- | --- | --- | --- | --- | --- | --- | --- |
| Total CUs | 33.9+10.8  [12.0, 55.8] | -0.2+0.2  [-0.5, 0.1] | -0.2+0.1  [-0.4, 0.1] | - |  | - |  |  | - |
| Left:Right CUs | 2.1+0.5  [1.1, 3.1]* | -0.01+0.01  [-0.03, 0.0001] | -0.01+0.004  [-0.02, 0.001] |  |  | - |  | - |  |
| Syll/Cus | 1.3_5.2  [-9.4, 12.0] | 0.1+0.1  [-0.1, 0.2] | 0.1+0.1  [0.002, 0.2]* | 0.001+0.001  [0.0001, 0.003] | -0.01+0.005  [-0.02, -0.001]* | -0.002+0.001  [-0.003, -0.001]* | 0.1+0.03  [0.1, 0.2]* |  | 0.001+0.0001  [0.0001, 0.002] |

Vol: Total lesioned volume.
